# TM9SF4 is a potential prognostic biomarker in hepatocellular carcinoma

**DOI:** 10.1007/s12672-025-02417-2

**Published:** 2025-04-23

**Authors:** Yahui Ma, Lingling Guo, Bo Zhang, Ting Wang, Qingchun Feng

**Affiliations:** 1 Department of General Surgery, Jiading District Central Hospital Affiliated Shanghai University of Medicine & Health Sciences, Shanghai, 201800 People’s Republic of China; 2 Department of Radiation Oncology, Peking University Shenzhen Hospital, Shenzhen, 518036 China; 3 Shanghai Key Laboratory for Cancer Systems Regulation and Clinical Translation, Shanghai, 201800 People’s Republic of China; 4 Department of Hepatobiliary and Pancreatic Surgery, Peking University Shenzhen Hospital, Shenzhen, 518036 China

**Keywords:** Hepatocellular carcinoma, TM9SF4, Prognosis prediction, Cell cycle

## Abstract

**Background:**

The transmembrane 9 superfamily protein member 4 (TM9SF4) is a transmembrane protein upregulated in multiple cancers; however, its role in hepatocellular carcinoma (HCC) remains unknown.

**Methods:**

The Cancer Genome Atlas (TCGA), Genotype-Tissue Expression (GTEx) and International Cancer Genome Consortium (ICGC) databases were utilized to investigate the differential expression of TM9SF4 in HCC and tumor tissues. The prognostic and value of TM9SF4 in HCC was evaluated using Kaplan–Meier analysis, Cox regression, and receiver operating characteristic (ROC) curve analyses. The expression pattern and prognostic value of TM9SF4 was further verified using immunohistochemical (IHC) examination of 87 pairs of HCC clinical specimens. A nomogram was constructed by combining TM9SF4 expression and clinicopathological parameters to predict prognosis for individual patient. Additionally, gene set enrichment analysis (GSEA) was performed to identify key pathways related to TM9SF4.

**Results:**

TM9SF4 expression was upregulated in the HCC tissues. High expression of TM9SF4 was significantly associated with advanced T stage, histological grade, and worse survival. Multivariable Cox analysis revealed that TM9SF4 expression was an independent factor for overall survival. The nomogram by incorporating the TM9SF4 and T stage showed good performance in predicting prognosis. Moreover, GSEA analysis revealed that TM9SF4 was functionally involved in pathways associated with the cell cycle.

**Conclusions:**

These findings suggest that TM9SF4 is a promising biomarker with prognostic potential and functional significance in HCC.

**Supplementary Information:**

The online version contains supplementary material available at 10.1007/s12672-025-02417-2.

## Introduction

Liver cancer is the sixth most commonly diagnosed cancer and the third leading cause of cancer-related death globally, with an estimated 0.86 million new cases and an estimated 0.76 million deaths worldwide in 2022 [1]. Hepatocellular carcinoma (HCC) is the most common prevalent liver malignancy, accounting for 85% of liver cancers. Surgical resection remains the primary method for curing HCC. However, HCC has a high recurrence rate post-surgery, with 5-year recurrence rates are up to 70%, and the 5-year overall survival (OS) rate is only approximately 25%–55% [2–4]. Currently, the molecular mechanisms and development of HCC are still unknown, and traditional pathological features are insufficient for accurate prediction of HCC prognosis. Therefore, identifying novel biomarkers and molecular targets is crucial for the diagnosis, treatment and prognosis of patients with HCC [5–8].

The transmembrane 9 superfamily protein member 4 (TM9SF4) is one of the members of the TM9SF protein family characterized by a long hydrophilic N terminus and nine putative transmembrane domains in the C-terminal region [9]. TM9SF4 have been shown to play an important role in phagocytosis, autophagy and cancer cell cannibalistic activity [9,10]. TM9SF4 is found to be upregulated in a variety of tumors, including melanoma, colon cancer cells, ovarian cancer cells, breast cancer, gastric cancer and gastrointestinal tumors [11–15]. In addition, the knockdown of TM9SF4 significantly reduces cell proliferation, migration and invasion of colon cancer and ovarian cancer cells7. In nasopharyngeal carcinoma, TM9SF4 promotes tumor cell proliferation through affecting GUSBP11/miR-1226-3p/TM9SF4 axis [16]. Yu et al. demonstrate that TM9SF4 promotes prostate cancer cell anoikis-resistance and metastasis [17]. These findings indicate that TM9SF4 overexpression may contribute to tumor development. However, the role and potential clinical value of TM9SF4 in HCC remain largely unknown.

In this study, we investigated the relationship between TM9SF4 expression and the clinical characteristics of patients with HCC, as well as the potential prognostic value and possible biological functions of TM9SF4 using TCGA data. Furthermore, we verified its expression through immunohistochemical examination of 87 paired HCC and normal tissues. By combining TM9SF4 expression and clinical characteristics, we constructed and validated a nomogram to predict prognosis in HCC for clinical use.

## Methods

### Clinical specimens and immunohistochemistry

In this retrospective study, we utilized an HCC tissue microarray (TMA, HLivH180Su10) from Outdo BioTech (Shanghai, China). The HLivH180Su10 contained 87 HCC tissues and 87 paired adjacent tissues, which was used as the validation cohort for the detection of TM9SF4 expression. Patient baseline information, including age, sex, liver cirrhosis, tumor size, tumor differentiation grade, lymph node metastasis, distant metastasis, TNM stage and follow-up data (follow-up duration and survival status), were also provided by Outdo BioTech (Shanghai, China). Ethical approval for the study of the TMA was granted by the Clinical Research Ethics Committee, Outdo BioTech (Shanghai, China). All patients met the following inclusion criteria: (1) histologically diagnosed HCC and treated with curative surgery, (2) no history of other malignancies and (3) clinicopathological and follow-up information were available.

The primary antibody was anti-TM9SF4 (1:200, Proteintech, Cat#25,595–1-AP). Immunohistochemistry (IHC) staining was performed on the TMA of HCC tissues according to the standardized procedure, using normal liver tissue as a control. The stained TMA was scanned using Pannoramic 250 Flash Slide Scanners. Then the TM9SF4 staining results were independently assessed by two pathologists. Immunohistochemistry score (H-score) was calculated for every sample by multiplying the intensity of TM9SF4 staining and the percentage of stained cells. The staining intensity was scored visually and stratified as follows: 0 (negative); 1 (weakly positive); 2 (moderately positive) and 3 (strongly positive).

### Data collection and analysis

We obtained mRNA levels of TM9SF4 in pan-cancer and corresponding normal tissues from the TCGAand the GTEx database. The ICGC LIHC dataset was also downloaded and applied as validation (ICGC, https://dcc.icgc.org/, release 28, accessed on 7 May 2024). The differences in TM9SF4 mRNA expression between tumor tissues and normal tissues were compared using Wilcoxon test.

### TM9SF4 expression correlates with prognosis and clinicopathological characteristics

Based on the median TM9SF4 expression, the patients were divided into low TM9SF4 expression and high TM9SF4 expression groups. The differences in the survival of the high and low TM9SF4 expression groups were evaluated using the Kaplan–Meier method and compared via log-rank tests. Using Wilcoxon rank sum test, we explore the relationship between TM9SF4 expression and clinicopathological characteristics. In addition, the TM9SF4 expression and clinicopathological characteristics were incorporated into the univariate Cox regression analyses for OS, and variables with P < 0.05 were selected in multivariate Cox regression analyses. The backwards stepwise regression was utilized to detect the independent predictors. A predictive nomogram including the independent predictors were developed to predict OS. The discrimination of the predictive nomogram was quantified using the area under the receiver operating characteristic curve (AUROC). The calibration curve was generated to compare the estimated survival probabilities with the actual probabilities. Decision curve analysis (DCA) was used to assess the clinical usefulness.

### Functional enrichment analysis

Differential gene expression analysis was performing using “limma” package. Genes that had an FDR < 0.05 and a |log2 FC|> 1 were identified as DEGs. Gene Oncology (GO) and KEGG (Kyoto Encyclopedia of Genes and Genomes) enhancement analysis were conducted for DEGs using “clusterProfiler” package. Gene Set Enrichment Analysis (GSEA) analysis was performed to investigate the potential biological differences between TM9SF4-high and TM9SF4-low group based on reference gene sets. The gene set “h.all.v7.4.symbols.gmt” was chosen for GSEA analysis. False discovery rate (FDR) < 0.25, P < 0.05, and normalized enrichment score (NES) > 1 was defined as statistically significant.

### Statistical analyses

Continuous variables were analyzed using the *t* test, and categorical variables were analyzed using the χ2 test. Survival curves were generated using the Kaplan–Meier method and compared using the log-rank test. Univariate and multivariate Cox regression were used to construct a Cox proportional risk model. The Wilcoxon rank-sum test was employed to analyze the differences between two groups. The diagnostic value of TM9SF4 was evaluated through receiver operating characteristic (ROC) analysis using the ‘pROC’ package. The area under the curves (AUCs) of the time-dependent ROC curves were calculated to assess the prognostic value of predictive models. Statistical analyses were performed using R 4.0.3 (R Project for Statistical Computing, Vienna, Austria, www.r-project.org). Differences with a 2-sided *P* < 0.05 were considered statistically significant.

## Results

### TM9SF4 expression in HCC

**Fig. 1:**
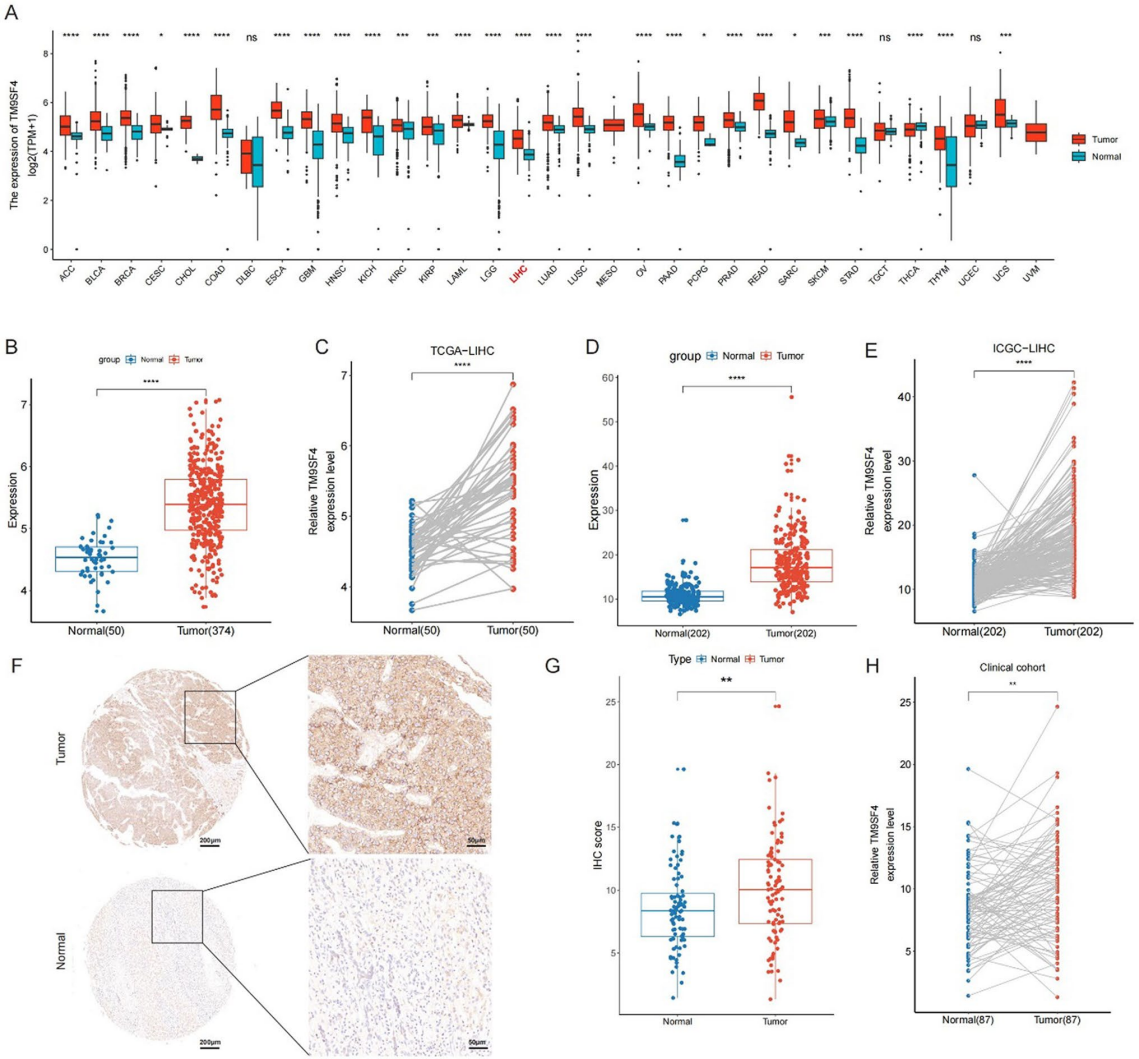
High expression level of TM9SF4 in pan-cancer and KIRC tumor tissues. **A** TM9SF4 expression in pan-cancer from TCGA and GTEx database. TM9SF4 expression in **B** unpaired and **C** paired samples from the TCGA-LIHC database. Expression of TM9SF4 in the **D** unpaired and **E** paired samples from the ICGC-LIHC databases. **F** Comparison of the protein levels of TM9SF4 between HCC and normal liver tissue samples via IHC. IHC score of TM9SF4 in **G** unpaired and **H** paired tissue microarray samples (n = 88). ns, *P* ≥ 0.05; * *P* < 0.05; ** *P* < 0.01; *** *P* < 0.001; **** *P* < 0.001

We first evaluated TM9SF4 expression in pan-cancer using data from TCGA and GTEx. The results showed that TM9SF4 was upregulated in various type of cancers, including HCC (Fig. 1A). In both paired samples and unpaired samples of the TCGA-LIHC database (Fig. 1B, C), the expression of TM9SF4 was significantly higher in tumor tissues compared with normal tissues (*P* < 0.05). These results were further validated using the ICGC-LIHC cohort (Fig. 1D, E). Clinical specimens confirmed the upregulation of TM9 FS4 in HCC (Fig. 1F). The IHC score of tumor tissue in the tissue microarray was greater than that of normal liver tissue (Fig. 1G, H). The receiver operating characteristics (ROC) curve also revealed TM9SF4 had a good diagnostic value (Supplementary Fig. 1).

### Association between TM9SF4 and clinical characteristics

**Fig. 2:**
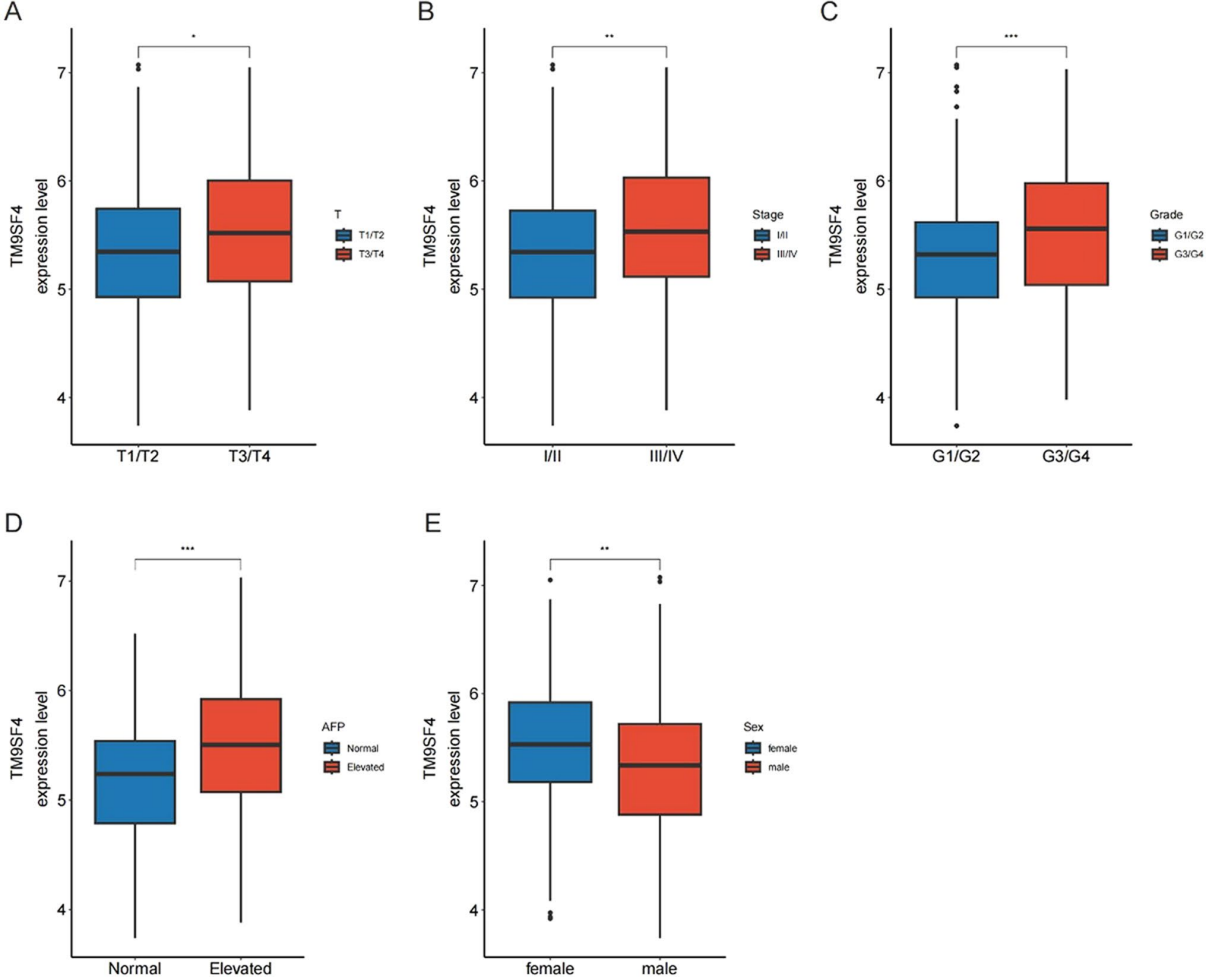
The expression of TM9SF4 was related with various clinicopathological factors. TM9SF4 expression in HCC according to **A** T stage, **B** TNM stage, **C** differentiation grade, **D** AFP levels and **E** age. ns, *P* ≥ 0.05; * *P* < 0.05; ** *P* < 0.01; *** *P* < 0.001; **** *P* < 0.001

In TCGA cohort, patients were categorized into high- and low-expression groups according to the median value of TM9SF4 expression, and the clinical characteristics are summarized in Table 1. We then analyzed the correlation among clinical characteristics and TM9SF4 expression levels in HCC. The results showed that higher T stage, TNM stage, AFP levels and worse differentiation grade had greater TM9SF4 expression, which indicated that TM9SF4 expression may influence cancer development and metastasis (Fig. 2A–D). We also found that female patients expressed higher TM9SF4 than male patients (Fig. 2F).

**Table 1:** The baseline information of HCC patients in TCGA cohort

Characteristics	High expression of TM9SF4 (N = 171)	Low expression of TM9SF4 (N = 172)	*P*
Age	58.2 (13.5)	60.0 (13.1)	0.203
Sex			0.013
Female	65 (38.0%)	43 (25.0%)	
Male	106 (62.0%)	129 (75.0%)	
T stage			0.117
T1	76 (44.4%)	97 (56.4%)	
T2	46 (26.9%)	40 (23.3%)	
T3	42 (24.6%)	32 (18.6%)	
T4	7 (4.09%)	3 (1.74%)	
N stage			0.014
N0	134 (78.8%)	116 (67.4%)	
N1	3 (1.76%)	1 (0.58%)	
NX	33 (19.4%)	55 (32.0%)	
M stage			0.415
M0	136 (79.5%)	126 (73.3%)	
M1	1 (0.58%)	2 (1.16%)	
MX	34 (19.9%)	44 (25.6%)	
TNM stage			0.052
I	75 (43.9%)	96 (55.8%)	
II	45 (26.3%)	40 (23.3%)	
III	50 (29.2%)	33 (19.2%)	
IV	1 (0.58%)	3 (1.74%)	
Differentiation grade			0.008
G1	17 (10.1%)	28 (16.3%)	
G2	73 (43.2%)	94 (54.7%)	
G3	72 (42.6%)	45 (26.2%)	
G4	7 (4.14%)	5 (2.91%)	
AFP^a^			< 0.001
Elevated	72 (57.6%)	48 (34.5%)	
Normal	53 (42.4%)	91 (65.5%)	

Categorical variables are compared using a two-sided χ2 test

*AFP* Alpha-fetoprotein, *TNM* tumor-node-metastasis

^a^ For AFP, elevated indicates 400 μg/L or greater

### Prognostic value of TM9SF4 in HCC

**Fig. 3:**
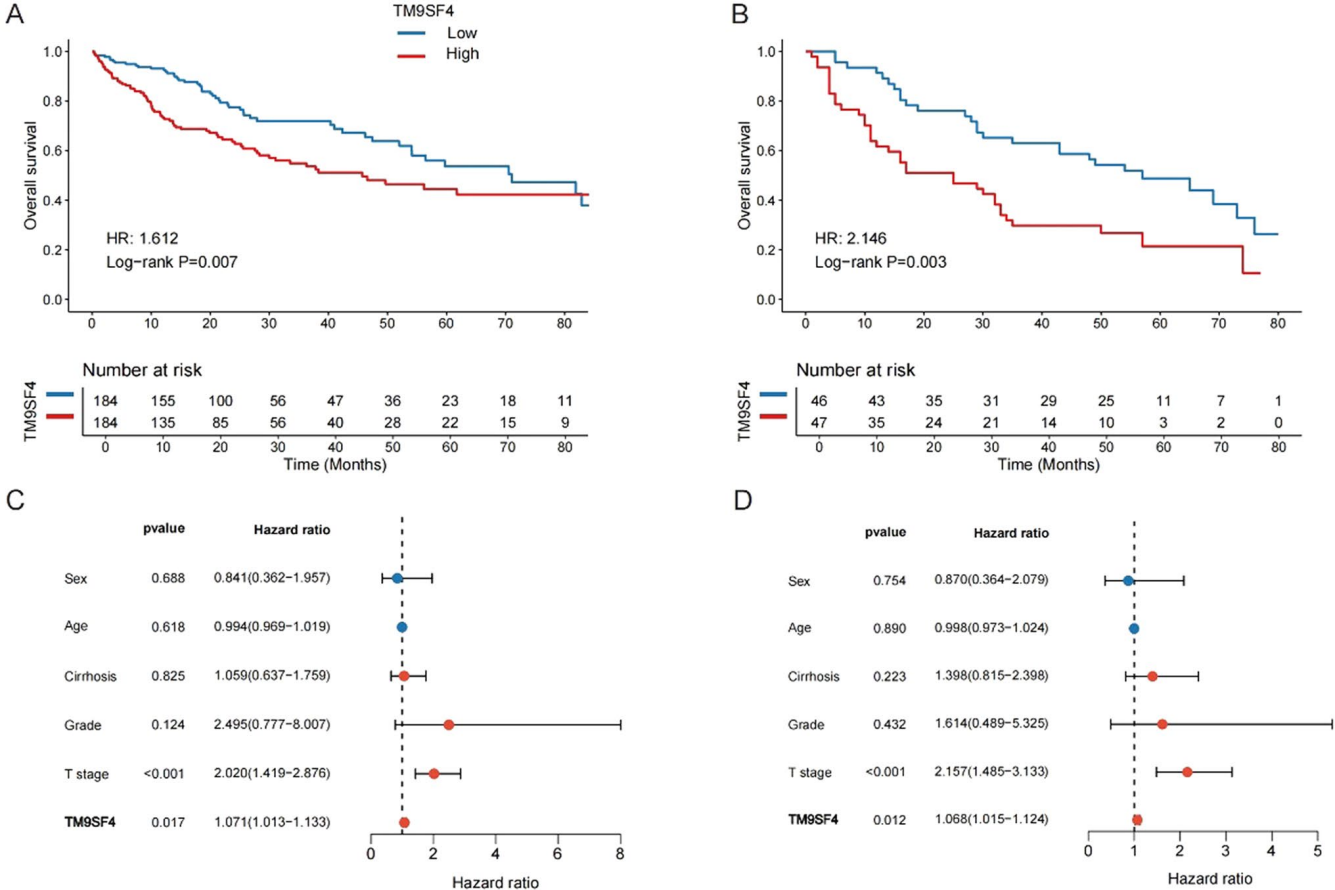
Prognostic value of TM9SF4 in HCC. Kaplan–Meier curve of TM9SF4 in **A** TCGA-LIHC cohort and **B** clinical cohort. **C**, **D** Univariate and multivariate Cox regression analyses indicating the effect of the IHC score of TM9SF4 on survival in the tissue microarray cohort

We explored the association between the expression of TM9SF4 with patients’ survival using Kaplan–Meier survival analysis in multiple HCC cohorts. Based on the median value of TM9SF4, patients in TCGA cohort were divided into TM9SF4-high and TM9SF4-low group. The results showed that patients with TM9SF4 overexpression had significant shorter survival than those with low TM9SF4 expression in both TCGA (HR: 1.162, 95%CI, 1.137–2.284, *P* = 0.007, Fig. 3A) and ICGC cohorts (HR: 2.287, 95%CI, 1.196–4.375, *P* = 0.012, Supplementary Fig. 2). In addition, Kaplan–Meier survival analysis of clinical cohort demonstrated that low TM9SF4 expression survived longer than those with high TM9SF4 expression (HR: 2.146, 95%CI, 1.292–3.562, Fig. 3B). Furthermore, we performed univariate and multivariate Cox regression analyses in clinical cohort (Fig. 3C, D). The findings of univariate analysis showed that T stage and IHC score of TM9SF4 were associated with the survival of HCC. The multivariate analysis further indicated that T stage and TM9SF4 expression were independent prognostic predictors for HCC.

### TM9SF4 nomogram construction and validation

**Fig. 4:**
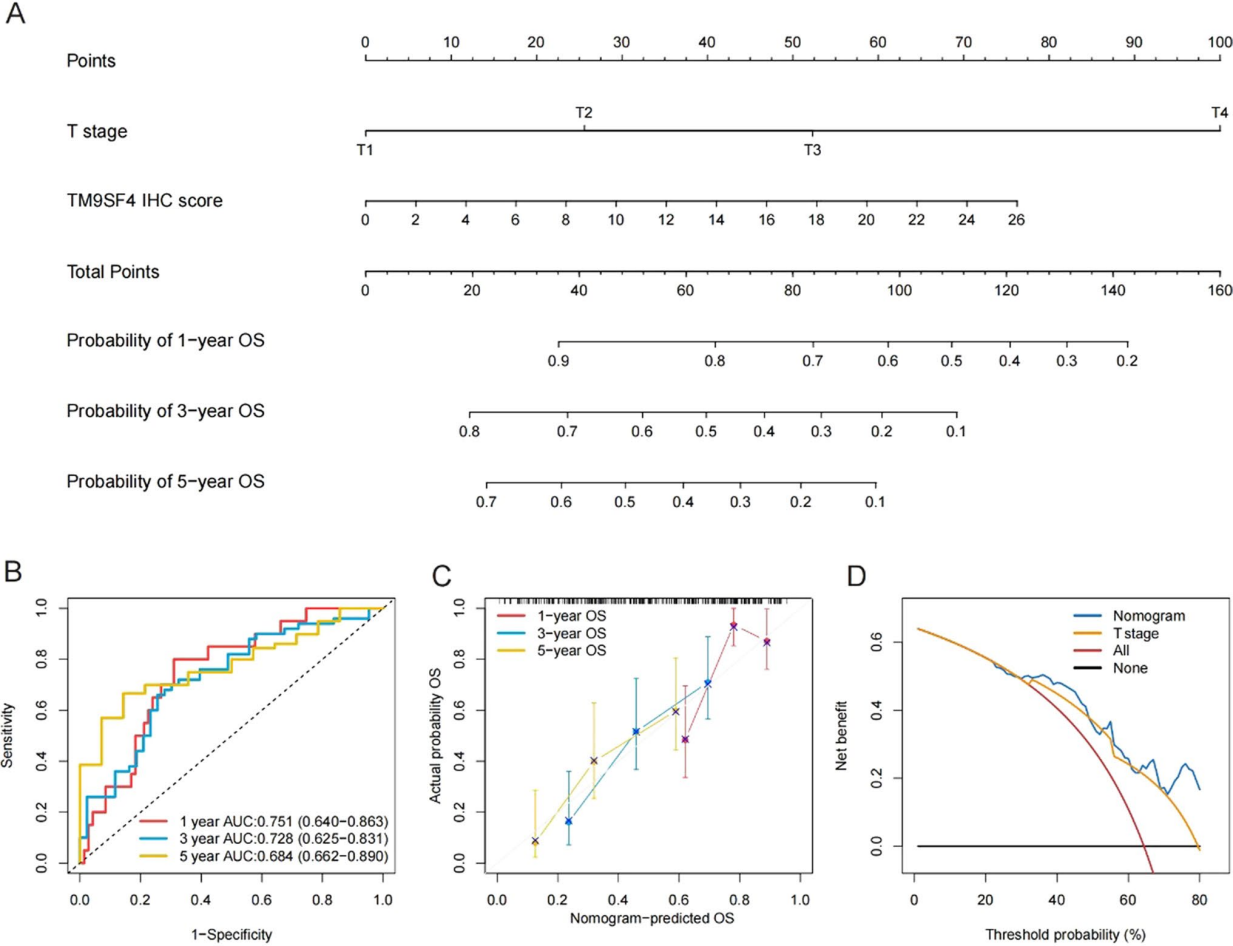
Construction and evaluation of Nomogram based on TM9SF4. **A** Nomogram for predicting OS in tissue microarray cohort. **B** Time-dependent ROC curves of the nomogram for predicting 1-, 3- and 5-year OS in the tissue microarray cohort. **C** Calibration curves of the nomogram for predicting 1-, 3- and 5-year OS in the tissue microarray cohort. **D** Decision curve analysis of OS for nomogram in tissue microarray cohort

Based on the multivariate Cox regression analysis, we constructed a predictive nomogram by integrating T stage and TM9SF4 expression to estimate the probability of OS in patients with HCC (Fig. 4A). The nomogram demonstrated satisfactory performance for prognosis estimation, with a concordance index of 0.700 (95% CI, 0.627–0.773) for OS. In addition, the time dependent ROC curve of the nomogram at 1, 3 and 5 years produced an area under the receiver operating characteristic curve (AUROC) of 0.751 (95% CI: 0.640–0.863), 0.728 (95% CI: 0.625 − 0.831) and 0.684 (95% CI: 0.662 − 0.890), respectively (Fig. 4B). Then, we validated this nomogram in TCGA cohort. The AUROCs of the nomogram at 1, 3 and 5 years were 0.731 (95% CI: 0.658 − 0.804), 0.709 (95% CI: 0.638 − 0.780) and 0.672 (95% CI: 0.582 − 0.763), respectively (Supplementary Fig. 3 A). The calibration curves showed good agreement between the nomogram-estimated survival outcomes and the actual survival outcomes at 1-, 3-, and 5-year prognosis in both cohorts (Fig. 4C, Supplementary Fig. 3B). Finally, the decision curve analysis revealed that using the nomogram to predict OS provided more net benefits than using the treat all scheme or treat none scheme in both clinical and TCGA cohorts, indicating that the nomogram was clinically applicable (Fig. 4D, Supplementary Fig. 3 C).

### Potential functions of TM9SF4 in HCC

**Fig. 5:**
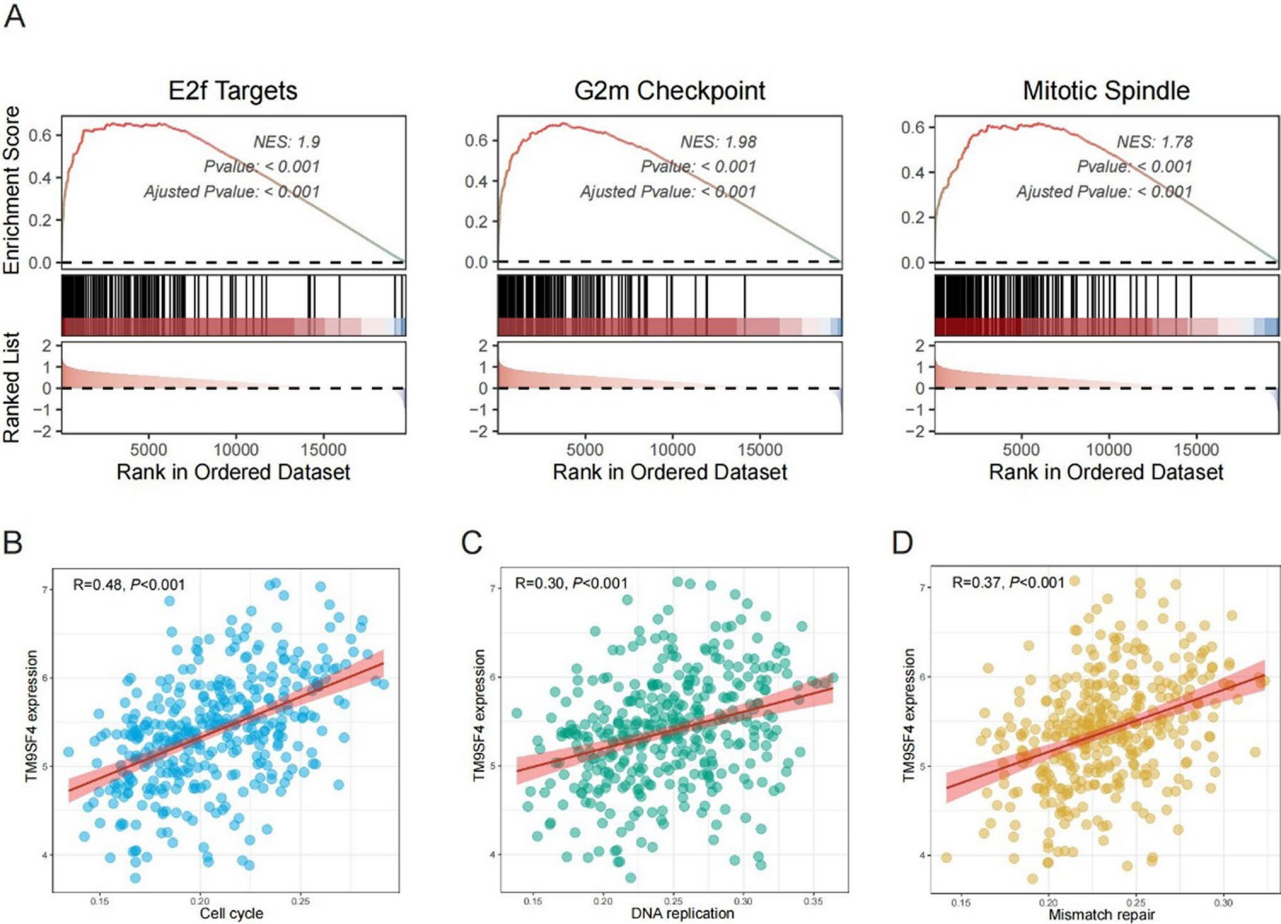
Functional Enrichment Analysis of TM9SF4 Expression in HCC. **A** GSEA analysis indicated that high expression of TM9SF4 was significantly correlated with E2 F Targets, G2M checkpoint, Mitotic Spindle and Protein secretion pathways in TCGA-LIHC dataset. TM9SF4 expression correlated with **B** cell cycle, **C** DNA replication and **D** mismatch repair signature

To elucidate the underlying mechanism of TM9SF4 in HCC, we performed GSEA using the TCGA database to identify significantly activated pathways in patients with high TM9SF4 expression compared to those with low TM9SF4 expression. Our results revealed that 13 pathways, including E2 F targets, G2M checkpoint and mitotic spindles, were significantly activated in patients with TM9SF4 overexpression (Fig. 5A, NES > 1.5, FDR < 0.25, and *P* < 0.001). Besides, we calculated various signatures covering tumor microenvironment, metabolic pathways, tumor intrinsic pathways via ‘IOBR’ packages and assessed their correlation with TM9SF4 expression^18^. We found that TM9SF4 was significantly positively correlated with cell cycle (Fig. 5B, R = 0.48, *P* < 0.001), DNA replication (Fig. 5C, R = 0.30, *P* < 0.001) and mismatch repair (Fig. 5D, R = 0.37, *P* < 0.001). Taken together, TM9SF4 may promote tumor progression in HCC by influencing cell cycle and DNA damage repair-related pathways.

## Discussion

TM9SF4 is an evolutionarily conserved transmembrane protein that is functionally involved in phagocytosis, autophagy and cancer cell cannibalistic activity. TM9SF4 is upregulated in various cancers and is often associated with poor prognosis. However, further investigation is warranted to elucidate the correlation between TM9SF4 expression and clinical characteristics and survival, thereby clarifying its implications for prognosis prediction and management of HCC.

In this study, we performed a bioinformatics analysis using TCGA database and found that TM9SF4 was upregulated in HCC. We also observed a significant correlation between TM9SF4 expression, AFP levels, and the histological grade of patients with HCC. These findings indicated that TM9SF4 may play a vital role in the occurrence and development of HCC. In addition, we verified the higher expression of TM9SF4 in tumor by IHC in tumor and normal tissue of 87 HCC patients from our clinical cohort. The expression of TM9SF4 in the T3-4 stages, poor differentiation grade and TNM stage III-IV was considerably greater than that in the T1-2, well differentiation grade and TNM I-II stage (*P* < 0.05), suggesting that high TM9SF4 expression was strongly connected to the incidence, development, metastasis and invasion in HCC. We then investigate the prognostic value of TM9SF4. The results showed that high expression of TM9SF4 was associated with worse prognosis in both TCGA, ICGC and clinical cohort.

Despite its limited performance, the TNM staging system remains one of the cornerstones for predicting the prognosis of patients with HCC. In our clinical cohort, we found that T stage was an independent predictor for prognosis via univariate and multivariate Cox regression. Importantly, the TM9SF4 expression was also identified as the independent risk factor for prognosis of HCC. Furthermore, by combining the TM9SF4 expression and T stage, we developed and validated a nomogram to predict prognosis for individual patient. These results indicate that the TM9SF4 provide complementary information about the prognosis of HCC.

From the perspective of clinicians, this nomogram is practicable for clinical application. On the one hand, T stage is routinely used characteristic in the clinic and is accessible during the perioperative period. On the other hand, the expression of TM9SF4 is easy to obtain from IHC of tumor tissues. Therefore, clinicians can easily perform the risk stratification and patients with worse prognosis could be distinguished, and more personalized interventions could be performed.

To elucidate the functional role of TM9SF4 in the development of HCC, we performed GSEA analysis in TCGA database. Our results showed that the cell cycle-related pathways, including E2 F targets, G2M checkpoint and mitotic spindles, were significantly activated in tumors with high TM9SF4 expression. In addition, we found that TM9SF4 was positively correlated with cell cycle, DNA replication and mismatch repair. These findings underscore the role of TM9SF4 in maintaining genomic stability and promoting cell proliferation.

There are some limitations in our study. First, given the retrospective design, our study was not free from inherent biases. Further validation in prospective study incorporating more external cohorts is warranted to test the prognostic value of TM9SF4. Second, in vitro and in vivo experiments are needed to investigate the underlying mechanism of TM9SF4 with cell cycle and DNA repair.

## Conclusions

In this study, we found that that TM9SF4 overexpression is associated with poor prognosis in HCC. By integrating the TM9SF4 expression with clinicopathological characteristics, we developed and validated a nomogram, which improved the prediction of the HCC prognosis compared to the clinicopathological characteristics alone. In addition, upregulation of TM9SF4 is positively correlated with cell cycle, DNA replication and repair. These findings highlight the oncogenic role of TM9SF4 and the nomogram developed in this study have the potential to improve prognosis prediction and guide individualized management in HCC.

## Supplementary Information

Additional file 1.

## Data Availability

The datasets used and/or analysed during the current study are available from the corresponding author on reasonable request.
