# Supplementary material for: TM9SF4 is a potential prognostic biomarker in hepatocellular carcinoma
Source: Discov Oncol. 2025 Apr 23;16:594. doi: 10.1007/s12672-025-02417-2 (PMC12018652; doi:10.1007/s12672-025-02417-2)
Supplement: Supplementary file 1 — Additional file 1. [file 12672_2025_2417_MOESM1_ESM.docx]

**Supplementary Figures**


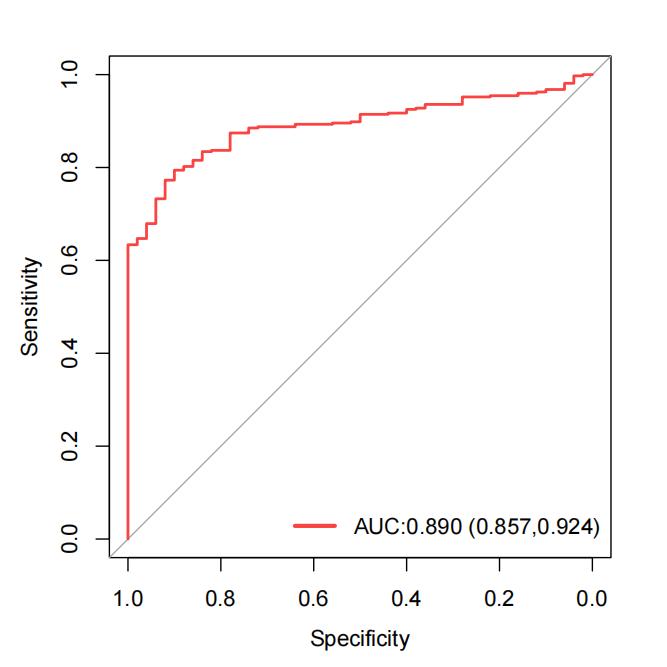


Supplementary Figure 1. The receiver operating characteristic (ROC) curves of TM9SF4.

**
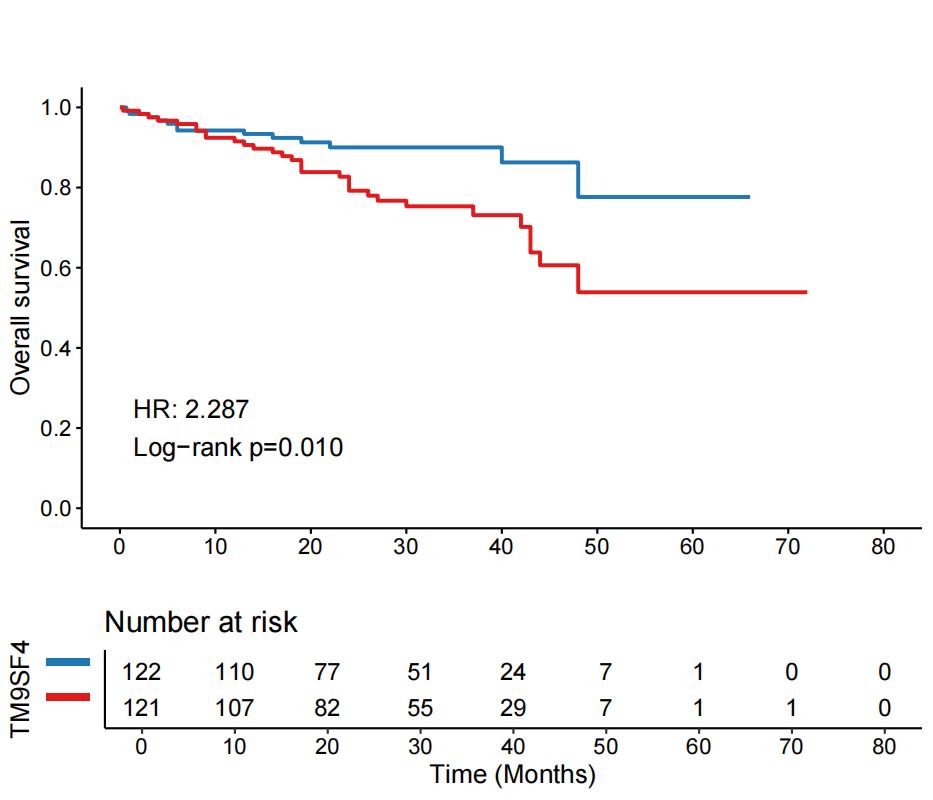
**

Supplementary Figure 2. The Kaplan-Meier curve of TM9SF4 in ICGC-LIHC cohort.


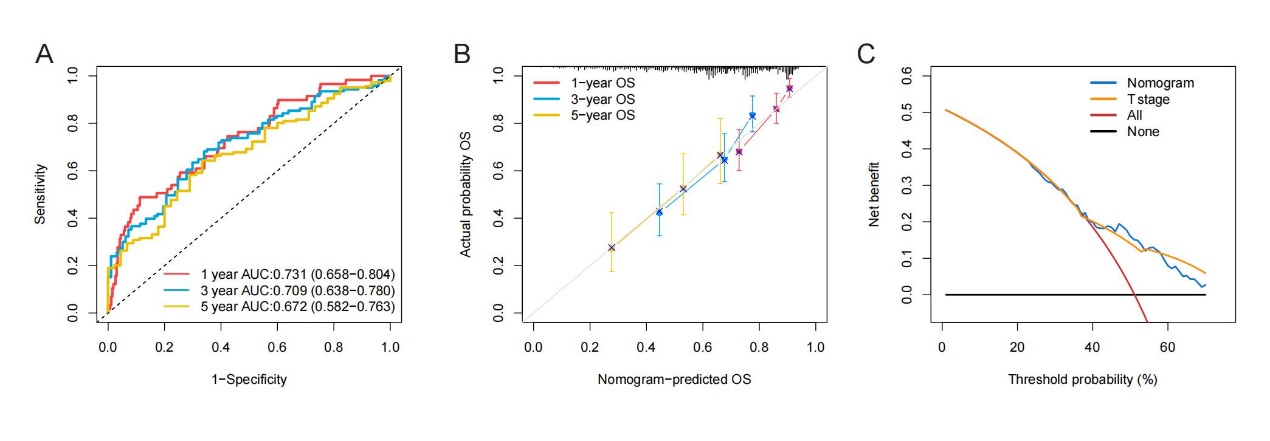


Supplementary Figure 3 Validation of Nomogram based on TM9SF4.

(A) Time-dependent ROC curves of the nomogram for predicting 1-, 3- and 5-year OS in TCGA cohort. (B) Calibration curves of the nomogram for predicting 1-, 3- and 5-year OS in TCGA cohort. (C) Decision curve analysis of OS for nomogram in TCGA cohort.
